# Determining the Effects of High Intensity Ultrasound on the Reduction of Microbes in Milk and Orange Juice Using Response Surface Methodology

**DOI:** 10.1155/2015/350719

**Published:** 2015-05-19

**Authors:** Balasubramanian Ganesan, Silvana Martini, Jonathan Solorio, Marie K. Walsh

**Affiliations:** ^1^Western Dairy Center, Utah State University, 8700 Old Main Hill, Logan, UT 84322-8700, USA; ^2^Department of Nutrition, Dietetics, and Food Sciences, Utah State University, 8700 Old Main Hill, Logan, UT 84322-8700, USA

## Abstract

This study investigated the effects of high intensity ultrasound (temperature, amplitude, and time) on the inactivation of indigenous bacteria in pasteurized milk, *Bacillus atrophaeus* spores inoculated into sterile milk, and *Saccharomyces cerevisiae* inoculated into sterile orange juice using response surface methodology. The variables investigated were sonication temperature (range from 0 to 84°C), amplitude (range from 0 to 216 *μ*m), and time (range from 0.17 to 5 min) on the response, log microbe reduction. Data were analyzed by statistical analysis system software and three models were developed, each for bacteria, spore, and yeast reduction. Regression analysis identified sonication temperature and amplitude to be significant variables on microbe reduction. Optimization of the inactivation of microbes was found to be at 84.8°C, 216 *μ*m amplitude, and 5.8 min. In addition, the predicted log reductions of microbes at common processing conditions (72°C for 20 sec) using 216 *μ*m amplitude were computed. The experimental responses for bacteria, spore, and yeast reductions fell within the predicted levels, confirming the accuracy of the models.

## 1. Introduction

High intensity ultrasound (HIU) waves (also known as power ultrasound; intensity >1 W/cm^2^, frequency 20 kHz) generate acoustic cavitation, where micro gas bubbles grow and implode to generate localized hot spots and increased pressure. The conditions within these collapsing bubbles generate localized temperatures exceeding 5,500°C and pressures of up to 50 MPa [1, 2]. The bubble collapse results in a radiation of shockwaves that damages bacterial cell walls and cellular structural and functional components such as DNA by intracellular cavitation [1, 2]. Ultrasound is commonly used in food processing for emulsification, controlling the viscosity of food systems, and improving cleaning and flux during ultrafiltration and microfiltration [1, 2].

Numerous studies have demonstrated the effective reduction of bacterial cells via ultrasound combined with heat treatment (thermosonication) in milk [3–5] and bacteria and yeasts in fruit juices [6–9]. Ultrasound application inactivates* Escherichia coli* and* Listeria* (85 W/cm^2^, 35°C) [10] at a slower rate in whole and skim milk versus buffer, which suggests that different matrices influence bacterial reduction. Thermosonication (50°C) was synergistically more effective than ultrasound alone for the inactivation of* E. coli* and* Staphylococcus epidermidis* (60 W, 50°C) in human milk [3]. Thermosonication (63°C for 0–30 min) improved the shelf life of whole milk [4, 5] by reducing the number of mesophilic bacteria demonstrating its potential in improving milk quality and safety. Other studies have shown reductions in* E. coli*,* Salmonella enteritidis*, and the yeast* Saccharomyces cerevisiae* and* Pichia fermentans* in fruit juices (mango, grape, orange, and tomato) with the application of thermosonication [6, 7, 9, 11]. Many studies focused on the reduction of vegetative bacteria that are killed by pasteurization, whereas there is less information on the effects of thermosonication on thermo/mesophilic bacteria and spore reduction in foods.

Belgrader et al. [12] showed the disruption of* Bacillus subtilis* spores in a minisonicator using power levels from 40 to 60 W for 2 min. Also, >99% disruption of* Bacillus globigii* spores was achieved with the same conditions in a continuous-flow system in microliter volumes [13]. These studies demonstrated that spore disruption is possible on a small scale with sonication, but optimization of conditions is necessary for large-scale use.

Based on previous studies, sonication temperature, amplitude, and time are the key parameters for microbial reduction in liquid systems. This study used response surface methodology (RSM) to optimize conditions of temperature, amplitude, and sonication time for maximum microbial reduction of bacteria and spores in milk and yeast in orange juice by determining the significant variables and creating predictive models for microbe reduction. RSM offers a large amount of information from a small number of experiments and it is possible to observe the interaction effects of the independent variables on the response. RSM also allows predictive responses for processing conditions (temperature and time) that have industrial importance. A sonication process that is amenable to the current industrial temperature and time processing conditions, such as pasteurization, is more likely to gain recognition and acceptance due to less infrastructural costs in modifying the processing equipment.

The purpose of this study was to determine the effects of sonication temperature, amplitude, and time on the inactivation of indigenous bacteria in pasteurized milk,* Bacillus atrophaeus* spores inoculated into sterile milk, and* Saccharomyces cerevisiae* inoculated into sterile orange juice. Data were analyzed by RSM and significant variables were determined. Predictive models for microbe inactivation were created and the suitability of the models was verified by comparing experimental optimal and realistic conditions to the predicted model.

## 2. Materials and Methods

### 2.1. Sources of Milk and Juice

Pasteurized skim milk was obtained from Rosehill Dairy (Hyrum, UT) and the Gary Haight Richardson Dairy Products Laboratory at Utah State University (Logan, UT). Shelf stable orange juice was purchased locally (Minute Maid brand). For studying spore reduction, ultrahigh temperature-treated skim milk (UHT milk) was obtained from Gossner Foods (Logan, UT).

### 2.2. Microbial Growth

Pasteurized milk (initial cell count was 10^2^ CFU/mL) was held at room temperature for 24 h to achieve a level of approximately 7 × 10^7^ CFU/mL indigenous thermophilic microbes. Total aerobic bacteria from untreated or sonicated milk samples were enumerated on tryptic soy agar plates at 30°C for 24–48 h. For spore samples, UHT milk was inoculated with 10^5^ *Bacillus atrophaeus* spores/mL (NAMSA, Northwood, OH). For studying yeast reduction in orange juice,* Saccharomyces cerevisiae* 1015 was obtained from Presque Isle Cultures (Erie, PA) and grown in potato dextrose broth at 30°C for 48–72 h prior to inoculation into sterile juice to achieve an initial level of approximately 10^6^ CFU/mL.* S. cerevisiae* was enumerated from sonicated samples after plating on potato dextrose agar and incubating at 30°C for 48–72 h.

### 2.3. Sonication Equipment and Conditions

Ultrasound treatment was done according to Martini et al. [14]. Briefly, 6 mL of milk or orange juice was sonicated in a double-walled glass vessel connected to a water bath. An ultrasonic processor (Sonicator 3000, Misonix Inc., Farmingdale, NY) set at 500 W and 20 kHz with a 3.2 mm titanium microtip was used. The sonication times, temperature, and amplitudes used are listed in Table 1. Overheating of samples during sonication was prevented by keeping the temperature in the sonication chamber constant using a water bath. The acoustic power obtained for each amplitude in each sample was calculated as described below.

### 2.4. Calculation of Acoustic Power

Acoustic power is determined by a calorimetric technique that measures the change in temperature in a known volume of sample at various ultrasonic amplitudes. Acoustic power delivered to the samples was calculated as described by Martini et al. [14] and Jambrak et al. [15], using the equation below:

(1) $$\begin{array}{c} P=M\times C_{p}\times \frac{dT}{dt}, \end{array}$$

where *P* is the acoustic power (W), *M* is the mass of sonicated sample (g), *C*_*p*_ is the specific heat capacity of the medium at constant pressure (J/g/K), and *dT*/*dt* is the increase in temperature during sonication (K/min). The specific heat capacity of the milk and orange juice (kJ/kg°C) used was determined by differential scanning calorimetry according to Martini et al. [14].

### 2.5. Experimental Design

To explore the effects of independent variables on the response, a RSM design (Roquemore R311A hybrid, SAS, 9.4, The SAS Institute, Cary, NC) with three variables (*X*_1_ = time (range from 0.17 to 5 min), *X*_2_ = temperature (range from 10 to 84°C), and *X*_3_ = sonication amplitude (range from 0 to 216 *μ*m)) was performed. The response variable was log_10_ reduction (*Y*_1_) of microbes compared to unsonicated controls. The design consisted of 11 experimental points (Table 1) that were conducted in duplicate. The coded values were low (−1.4), central (0), and high (1.4).

### 2.6. Statistical Analysis

The response surface regression (RSREG) procedure of statistical analysis was used to analyze the experimental data. Experimental data were fitted to a second-order polynomial model and regression coefficients were obtained. The generalized second-order polynomial model used in the response surface analysis is given below:

(2) $$\begin{array}{c} Y=\beta_{0}+\overset{3}{\underset{i=1}{\sum }}\beta_{i}X_{i}+\overset{3}{\underset{i=1}{\sum }}\beta_{ii}X_{i}^{2}+\sum \overset{3}{\underset{i<j=1}{\sum }}\beta_{ij}X_{i}X_{j}, \end{array}$$

where *β*_0_, *β*_*i*_, *β*_*ii*_, and *β*_*ij*_ are the regression coefficients for intercept, linear, quadratic, and interaction terms and *X*_*i*_ and *X*_*j*_ are the independent variables. Residual analysis was performed (box and scatter plots) and the identification of outliers and influential data points was done to confirm adequacy of the data. Validity of the polynomial model was tested with analysis of variance (ANOVA). The significances of all terms in the polynomial were judged statistically by computing the *F*-value at *p* = 0.05. Lack-of-fit significance, as well as *R* ^2^, and adjusted *R* ^2^ were evaluated for model accuracy. The design software was used to generate response surface plots while holding a variable constant in the second-order polynomial model while maximizing *Y*_1_. The ridge max option was used to compute the estimated ridge of maximum response for increasing radii from the center of the original design. Canonical analysis was conducted to determine the overall shape of the curve and to determine which variables(s) were the most influential.

### 2.7. Verification of Model

The predicted model was used to determine the optimal conditions for microbial reduction as well as estimate microbial reduction at common food processing conditions (72°C, 10 s, 20 s) with amplitude of 216 *μ*m. Experimental microbial reduction was compared to the predicted model to assess the accuracy of the predicted models.

### 2.8. Microbial Identification

Microbial colonies were isolated by plating serial dilutions of milk samples on tryptic soy agar (incubated at 30°C for 48 h) at two different times 3 months apart. From these plates, 10 colonies were randomly chosen for DNA-based identification. Briefly, bacterial colonies were grown in tryptic soy broth for 48 h. After growth was visually confirmed, cells were collected by centrifugation (12,000 ×g for 2 min) and DNA was extracted after physical lysis using glass beads and using phenol-chloroform-isoamyl alcohol extraction as described by Ganesan et al. [16]. The isolated DNA was further used for 16S ribosomal gene amplification by PCR using bacterial 16S rRNA gene primers (16S rRNA forward AGAGTTTGATCCTGGCTCAG and 16S rRNA reverse ACGGCTACCTTGTTACGACTT; Integrated DNA Technologies, Coralville, IA) and using a 2X PCR MasterMix (Thermo Fisher Scientific Inc., Pittsburgh, PA). Each final reaction mixture contained the following: template DNA, 1-2 *μ*g; MgCl_2_, 2.5 mM; primers, 10 pmol; and each dNTP, 0.4 mM. The amplification reaction was performed on a GeneAmp PCR system 2400 thermal cycler (Perkin Elmer, Waltham, MA) with initial enzyme activation at 95°C for 3 min, followed by 40 cycles of denaturation at 95°C for 30 s, annealing at 50°C for 30 s, and extension at 72°C for 1 min. The resulting PCR product was purified using a commercial kit (QiaQuick PCR purification kit, Qiagen, Valencia, CA) and submitted to USU's Center for Integrated BioSystems for single gene sequencing. Sequence results were compared against NCBI's nonredundant public databases using NCBI's BLAST software to provide species identification, which was assigned at ≥97% sequence identity to the nearest known reference database hits (*E*-value ≤10^5^).

## 3. Results

### 3.1. Statistical Analysis and Model Fitting

We investigated the effects of temperature, amplitude, and time on the inactivation of bacterial cells and spores in milk and yeast in orange juice using RSM. The log reductions of microbes with the treatments used are given in Table 1. A regression analysis (Table 2) was done to fit the mathematical models to the experimental data and the significance of each coefficient was determined using the *F*-test and *p* value. For bacterial reduction in milk, the significant variables, in order with the largest effect first, were temperature (*X*_2_), followed by *X*_2_ *∗X*_2_, amplitude (*X*_3_), and *X*_3_ *∗X*_3_. For spore reduction in milk, the significant variables, in order with the largest effect first, were, *X*_3_, *X*_2_, *X*_2_ *∗X*_2_, *X*_2_ *∗X*_3_, *X*_1_ (time) *∗X*_1_, and *X*_1_. For yeast reduction in orange juice, all variables without time were significant, with the largest effects consisting of *X*_2_, *X*_3_, *X*_2_ *∗X*_2_, *X*_2_ *∗X*_3_, and *X*_3_ *∗X*_3_ sequentially. The results suggest that temperature and amplitude were highly significant for the reduction of microbes, while time was only significant with spore reduction. The greatest log reductions of indigenous bacteria and spores in skim milk and yeast in orange juice are seen at the highest temperature (84.4°C), while low treatment conditions for temperature and amplitude lead to low log reductions (Table 1). The acoustic power is the actual power dissipated as heat in the treated system and varies based on the amplitudes used and the specific heat capacity of the liquid. The effects of acoustic power on microbe reduction are not obvious in Table 1 due to the different temperatures and times used for each sample.

Analysis of variance (ANOVA) for the models is given in Table 3. Hypothesis test on linear, quadratic, and cross product in ANOVA indicated that linear and quadratic regressions showed significant contribution to the models while cross product was also significant in the yeast and spore reduction models. The lack-of-fit error was insignificant for each model and the coefficients of determination (*R* ^2^) for each predictive model (86.52, 87.24, and 99.66, resp., for bacteria, spores, and yeast) were adequate, suggesting a good fit. Thus the response was sufficiently explained by the models.

**Figure 1:**
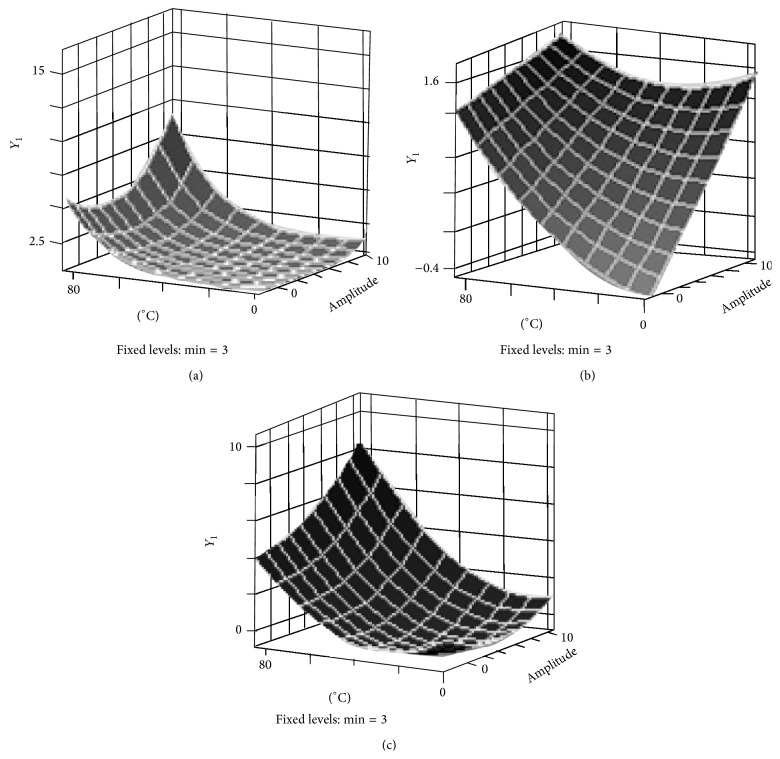
Response surface plots showing the optimization of sonication temperature and amplitude on the log reduction of indigenous bacteria (*Y*_1_) in milk (a), the log reduction of inoculated* Bacillus atrophaeus* spores (*Y*_1_) in milk (b), and the log reduction of inoculated* S. cerevisiae* (*Y*_1_) in orange juice (c).

RSM allows the identification of optimum values of temperature, amplitude, and time to maximize the response (*Y*_1_ or log reduction). Three-dimensional response surfaces for the graphical representation of the regression equations are shown in Figure 1. In the plots, temperature and amplitude were developed for maximum log reduction (*Y*_1_) while time was held constant. This was based on the significance of the independent variables given in Table 2. The maximum predicted log reductions were found at the highest temperature and amplitudes used. For spore reduction (Figure 1(b)) there was a linear increase in spore reduction with both an increase in temperature and amplitude, which agrees with the model analysis in Table 3. For yeast reduction (Figure 1(c)), temperature played a role higher than 40°C, while there was a steady increase in log reduction of yeast with an increase in amplitude. Similarly, yet less dramatic effects are seen for bacterial reduction in milk (Figure 1(a)). The predictive mathematical models are given in Table 4. For each model, canonical analysis demonstrated a minimum point as the stationary point and of the three variables the most influential factor was temperature which is consistent with the regression analysis.

### 3.2. Verification of Models

The suitability of the model equations for predicting the optimal and realistic responses was tested and the data is shown in Table 5. Maximum microbe reduction for each was predicted at the highest temperature (84.8°C) and amplitude (216 *μ*m) and longest sonication time (5.8 min) with predicted values of 15, 2.29, and 9.42 log reductions each for bacteria, spores, and yeast. The actual log reduction for spores was within the predicted range. Since the initial cell counts of microbes in milk and orange juice were below the predicted log reduction, the data shown is the inactivation of all bacteria and yeasts in the samples. Foods are rarely processed above 75°C; therefore, the realistic thermal processing conditions of 72°C and times of 10 and 20 sec were used in the SAS response calculator to obtain predicted log reductions. As seen in Table 5, the actual log reductions fell within predicted levels for the realistic processing conditions. Doubling sonication time from 10 to 20 seconds did not increase the predicted bacteria and yeast counts because the sonication time effect was not significant in these models.

### 3.3. Microbial Identification

Ten random colonies from plated milk samples were selected for identification by 16S rRNA gene sequencing. We found that the closest species matching the isolated colonies' DNA belonged to various species of bacteria such as* Pseudomonas*,* Lactococcus*,* Leuconostoc*,* Brochothrix*, and unknown Enterobacteriaceae members. The presence of spore-forming bacteria was not detected by our approach.

## 4. Discussion

Thermophilic bacteria, yeasts, and spores can survive the current pasteurization of fruit juices and milk [17, 18]. The presence of these microbes reduces product quality and shelf life due to production of acid, lipases, proteases, and off-flavors during storage [17, 19]. Additionally, the germination of spores is a concern in nonfat dry milk and skim milk powders because the powders themselves or food products formulated with them can have inferior quality and acceptability [17]. Pasteurization of milk and equivalent treatments for other liquid foods have been the most effective way of reducing microbial load while continuing to preserve freshness. Higher heat treatments such as ultrahigh temperature heating improve microbial quality at the expense of product freshness. Coupling ultrasound with pasteurization may aid in reducing the thermophilic microbes in fluid foods [19] extending the quality and shelf life of the products. Previous research has shown that no off-flavors were generated in 10% whey suspensions sonicated for 15 min using a power level of 3 W; therefore product freshness should be retained in solicited milk [20]. Factors that affect the inactivation of microbes by ultrasound are the temperature of treatment, amplitude of the ultrasonic waves, the exposure time, the type of microorganism, the volume of food to be processed, and the composition of the food.

A recent study by Herceg et al. [21] used RSM to investigate the effects of temperature, amplitude, and sonication time on the inactivation of* Staphylococcus aureus* and* E. coli* in milk. They showed that temperature, time, and amplitude were significant for microbe inactivation. In this study, we showed that temperature had the greatest effect, followed by amplitude, on microbial reduction. Previous studies have shown that inactivation of bacteria and spores increases with the amplitude of the sonication waves [22, 23]. The higher inactivation rate at greater amplitudes could be due to an increase in the number of bubbles undergoing cavitation [24].

This study showed that thermosonication was effective in reducing the bacterial load by at least 5  logs in milk at conditions of 72°C and 10 sec and at an amplitude of 216 *μ*m. Previous studies have also shown that there is a synergistic effect of heat and sonication [3, 5, 6, 21, 25] on the reduction of bacteria. The listed studies used temperatures greater than 40°C and our data is in agreement that temperatures greater than approximately 40°C with sonication are effective for bacteria reduction. Herceg et al. [21] stated that the synergy between heat and ultrasound reduces at temperatures greater than 60°C, turning into a cumulative effect.

There is conflicting data on the sensitivity of Gram-positive and Gram-negative bacteria with some studies stating that they are affected equally, while others showed that either one or the other was more sensitive [1, 2, 21], suggesting that either the Gram-positive bacterial cell wall or the Gram-negative bacterial cell membrane may be variably sensitive to ultrasound. We used indigenous bacteria present in pasteurized milk assuming they would be thermophiles. The identified bacteria in milk used here were both Gram-positive and Gram-negative and we did not determine whether there was differential reduction. Although these bacteria are not classified as thermophiles, they have been known to survive pasteurization conditions.

Studies on the inactivation of yeast in juices with sonication have shown a 7-log reduction of* S. cerevisiae* in grape juice at 60°C [7] and greater than a 5-log reduction of* Pichia fermentans* in tomato juice at 40°C [9]. Temperatures greater than 40°C were necessary for the inactivation of* S. cerevisiae* and the maximum inactivation of* S. cerevisiae* was observed at 60°C. Valero et al. [26] showed a 0.6-log reduction of indigenous yeast in orange juice at 88°C yet the conditions used by Khanal et al. [19] had no effect on indigenous yeasts (10°C, 8 min, and 89 *μ*m). Our results are in agreement in that temperatures greater than 40°C are needed for the inactivation of* S. cerevisiae*.

We showed a 1- to 2-log reduction of* Bacillus* spores at 72–85°C, which is significant, considering that ultrahigh temperature treatment (121–140°C) is the only established process for spore removal in milk in the food industry. Thermosonication is thus a viable approach for achieving microbial reduction greater than heat alone without going to ultrahigh processing temperatures. The design of an applicable process in pilot-scale fluid operations is a necessary step for confirming the benefits of thermosonication.

## 5. Conclusion

RSM was effective at estimating the effect of three independent variables on the log reduction of bacteria, spores, and yeast. Temperature and amplitude had highly significant effects on the response value as well as the quadratic of temperature. Optimum conditions for maximum log reduction were determined as well as the predicted log reductions as practical processing conditions. Experimental results were in the predicted ranges, verifying the accuracy of the models. Future research on verifying the accuracy of the models on a pilot scale using flow through thermosonication is necessary.

## Figures and Tables

**Table 1:** The response surface experimental design and results for the log reductions of bacteria and spores in milk and yeast in orange juice.

Run	Time (min)	Temp. (°C)	Amplitude (*μ*m)	Actual log reduction bacteria	Actual log reduction spores	Actual log reduction yeast	Acoustic power in milk (W)	Acoustic power in juice (W)
1	3	41	216	3.37	1.89	3.4	6.19	5.82
2	3	41	0	0.64	0.01	0	0	0
3	1	10	180	0.49	1.00	0	11.12	10.59
4	5	10	180	0.45	1.05	0	4.41	4.54
5	1	72	180	3.04	1.34	5.85	10.40	10.82
6	5	72	180	4.28	1.60	5.85	2.59	2.39
7	5	41	36	1.26	1.20	0.10	1.03	1.00
8	0.17	41	36	0.45	0.56	0.20	10.22	7.35
9	3	84	36	7.76	1.80	5.90	0.64	0.71
10	3	0	36	0.48	0.10	0	0.68	1.08
11	3	41	108	0.53	0.50	0.40	3.05	2.90

**Table 2:** Estimated regression model of the relationship between response variable (*Y*_1_) and independent variables (*X*_1_, *X*_2_, and *X*_3_).

	Bacteria in milk		Spores in milk		Yeasts in OJ	
	*F*-value	Prob. >*F*	*F*-value	Prob. > *F*	*F*-value	Prob. > *F*
*X*_1_ = time	3.84	0.0735	5.63	0.0352	0.18	0.6775
*X*_2_ = temperature	78.62	0.0001	53.58	0.0001	3622.7	0.0001
*X*_3_ = amplitude	9.50	0.0095	59.02	0.0001	692.53	0.0001
*X*_1_∗*X*_1_	0.28	0.6034	8.15	0.0145	1.06	0.3241
*X*_1_∗*X*_2_	0.46	0.5086	0.12	0.7315	0.00	1.0000
*X*_1_∗*X*_3_	1.87	0.1961	2.09	0.1741	0.18	0.6775
*X*_2_∗*X*_2_	13.24	0.0034	10.51	0.0070	627.98	0.0001
*X*_2_∗*X*_3_	0.025	0.8776	9.40	0.0098	106.64	0.0001
*X*_3_∗*X*_3_	6.75	0.0233	4.28	0.0607	81.85	0.0001

*X*_1_ = time (min), *X*_2_ = temperature (°C), *X*_3_ = amplitude, and *Y*_1_ = log reduction.

**Table 3:** Analysis of variance for the response surface quadratic model for log reductions of microbes.

	Bacteria in milk		Spores in milk		Yeast in orange juice	
	*F*-value	Prob. > *F*	*F*-value	Prob. > *F*	*F*-value	Prob. > *F*
Model	12.39	0.0001	15.83	0.0001	579.01	0.0001
Linear	30.66	0.0001	39.41	0.0001	1438.5	0.0001
Quadratic	5.75	0.0113	4.23	0.0295	262.92	0.0001
Cross product	1.61	0.5237	3.87	0.0379	35.61	0.0001
Lack-of-fit	12.40	0.1048	206.64	0.0901	15.92	0.1021

**Table 4:** The predicted mathematical models for bacteria, spore, and yeast reduction after ultrasound treatment.

Microorganism	Polynomial
Bacteria in milk	−0.85514 − 0.013031∗*X*_2_ − 0.124421∗*X*_3_ + 0.000553∗*X*_2_∗*X*_2_ + 0.022416∗*X*_3_∗*X*_3_
Spores in milk	−0.13737 − 0.14663∗*X*_1_ + 0.006844∗*X*_2_ + 0.172535∗*X*_3_ + 0.035885∗*X*_1_∗*X*_1_ + 0.000182∗*X*_2_∗*X*_2_ − 0.001798∗*X*_2_∗*X*_3_
Yeast in orange juice	0.491926 + 2.496654∗*X*_2_ + 1.091597∗*X*_3_ + 1.418168∗*X*_2_∗*X*_2_ + 0.605779∗*X*_2_∗*X*_3_ + 0.611709∗*X*_3_∗*X*_3_

*X*_1_ = time (min), *X*_2_ = temperature (°C), and *X*_3_ = amplitude.

**Table 5:** Comparison of the predicted and actual log microbial reductions in milk and juice attemperatures and times listed (all samples were run at 216 μm amplitude).

Time (min)	Temp. (°C)	Predicted log reduction (range)	Actual log reduction
Indigenous thermophilic bacteria in milk			
5.8	84.8	15 (12.2, 17.95)	6.54^1^
0.17 (10.2 sec)	72	5.85 (3.20, 8.51)	5.24
0.33 (20 sec)	72	5.85 (3.24, 8.47)	5.39

Spores in milk			
5.8	84.8	2.29 (1.52, 3.07)	2.01
0.17 (10.2 sec)	72	1.66 (1.09, 2.23)	1.56
0.33 (20 sec)	72	1.68 (1.17, 2.11)	1.66

*S. cerevisiae* in orange juice			
5.8	84.8	9.42 (9.12, 9.72)	6.57^1^
0.17 (10.2 sec)	72	6.72 (6.49, 6.94)	6.56^1^
0.33 (20 sec)	72	6.72 (6.49, 6.94)	6.57^1^

^1^The reduction represents the maximum amount of microbes in the sample.
